# Level of Mothers'/Caregivers' Healthcare-Seeking Behavior for Child's Diarrhea, Fever, and Respiratory Tract Infections and Associated Factors in Ethiopia: A Systematic Review and Meta-Analysis

**DOI:** 10.1155/2022/4053085

**Published:** 2022-07-18

**Authors:** Getachew Assefa Zenebe, Seblewongel Gebretsadik, Temesgen Muche, Daniel Sisay, Abinet Meno, Habtamu Endashaw Hareru, Berhanu Gidisa Debela

**Affiliations:** ^1^School of Public Health, College of Medicine and Health Sciences, Dilla University, Ethiopia; ^2^Department of Nutrition, School of Public Health, College of Medicine and Health Sciences, Dilla University, Ethiopia; ^3^School of Medicine, College of Medicine and Health Sciences, Dilla University, Ethiopia

## Abstract

**Objective:**

To assess the pooled prevalence of mothers' or caregivers' healthcare-seeking behavior for childhood diarrhea, fever, and respiratory tract infections and associated factors in Ethiopia. *Study Design*. Systematic review and meta-analysis.

**Methods:**

Literature searches were conducted through databases (Google Scholar, PubMed, CINHAL, ScienceDirect, HINARI, and gray literatures) from September 1 to 30, 2021, using key terms in accordance with the PRISMA guidelines. The characteristics of the original articles were described using text and tables. Heterogeneity among the reported prevalence of studies was checked by using a heterogeneity *χ*^2^ test and I^2^ test. Publication bias was examined by performing Egger's correlation and Begg's regression intercept tests at a 5% significant level. A random-effect model was employed to estimate the pooled prevalence of the outcome variable and its determinants in Ethiopia.

**Results:**

Of the total identified studies, 25 studies were included in the review, with a total of 29,993 study participants. The overall pooled prevalence of mothers' or caregivers' health-seeking behavior for childhood diarrhea, fever, and respiratory tract infections was 60.33% (95% CI: 50.14-70.52). The significant factors were residence (AOR = 3.06, 95% CI: 1.11–8.39), wealth index (AOR = 2.18, 95% CI: 1.92-2.48), perceived severity of illness (AOR = 2.7, 95% CI: 1.12–6.51), and knowledge of the illness (AOR = 1.95, 95% CI: 1.37–2.75).

**Conclusion:**

This review suggests that the overall pooled prevalence of mothers' or caregivers' HSB for childhood diarrhea, fever, and respiratory tract infections was 60.33%. Residence, wealth index, perceived severity of illness, and knowledge of the illness by mothers were the significant factors. Therefore, providing interventions by considering the above factors will improve the overall seeking behavior.

## 1. Introduction

In the last three decades, child mortality has decreased from 12.5 million in 1990 to 5.2 million in 2019. Although global progress has been made in reducing child mortality, around 5.3 million under-five children died in 2018, with almost half of these deaths occurring in sub-Saharan Africa [1]. Reports reveal that children in sub-Saharan Africa are more than 15 times more likely to die before age 5 than in high-income countries [2]. In Ethiopia, there were 67 deaths per 1000 live births in 2016 [3].

Morbidity contributes substantially to these deaths, as pneumonia, malaria, and diarrhea have been linked to about 29% of the global deaths of under-five children in 2018. In 2017, almost 1.6 million people died from diarrheal diseases, and more than 350,000 (57%) of those deaths were from malaria in children under-5 globally in 2017 [4, 5]. While malaria resulted in approximately 266,000 deaths of these children, diarrhea is responsible for 480,000 deaths of young children across the world in 2018 [6]. If care is sought early enough, the morbidity and mortality from these diseases can be reduced considerably. Globally, more than half of all early childhood complications and deaths are caused by illness, which can be avoided or treated with simple, low-cost interventions, and timely access to appropriate healthcare [7].

Healthcare-seeking behavior is an action taken by an individual in response to an internal and external stimulus to find a suitable solution after a child has a health problem [8]. Health-seeking behavior is a function of not only the accessibility of health facilities and other sources of healthcare but also the inspiration and capacity of individuals to seek medical treatment [8]. Inability to seek healthcare or delay in proper care of mothers in modern health facilities is a major cause of child death all over the world, especially in sub-Saharan Africa [9]. According to the WHO, child mortality and morbidity could be reduced by 20% if there was appropriate health-seeking behavior [8]. Therefore, the ability of caregivers to recognize and seek appropriate care for these childhood diarrhea, fever, and respiratory tract infections is an instrument in reducing child deaths in low- and middle-income countries, especially in Ethiopia [7, 10].

The magnitude of a mother's healthcare-seeking behavior for their childhood illness is different across countries and its regions [10–19]. A study conducted in developing countries showed that about 73.0% of caregivers sought care from a healthcare provider when their child was suffering from diarrhea, malaria, or pneumonia, with a median of 44.9% seeking care from appropriate providers. Care seeking was highest for pneumonia, with 91.3%, and lowest for diarrhea, with 68.5%. Seeking no care was most common for diarrhea (21.3%) and least common for malaria (8.1%). Appropriate care was sought most frequently for pneumonia (84.0%) and least frequently for malaria (42.5%) [10]. In addition, around 85% of women in sub-Saharan African countries sought healthcare for childhood illnesses, with the highest and lowest prevalence in Gabon (75.0%) and Zambia (92.6%), respectively [13].

In Ethiopia, the magnitude of a mother's healthcare-seeking behavior ranges from 27.2% to 90.6% [11, 15–24]. A study conducted in Addis Ababa, Ethiopia, revealed that the proportion of healthcare-seeking behavior of care-givers for childhood illnesses was 69.5% [17]. Another study conducted in Mekelle, Ethiopia, found that 72.5% of mothers who reported their children having diarrhea sought healthcare, with 75.9% seeking health in public healthcare facilities. 89.3% of those children who had severe diarrhea also sought healthcare facilities [18]. In addition, around 76.2% of mothers sought modern healthcare in Shire Town, Ethiopia [19].

In studies conducted in various countries, residence, severity of illness, caregiver education level, gender, socioeconomic status, cost of healthcare, knowledge of caregiver/mother for childhood illness, and perceived severity of illness were found to be common contributing factors for healthcare-seeking behavior [10, 12, 13, 17, 25].

Even though childhood diarrhea, fever, and respiratory tract infections are manageable successfully if recognized in time, facilitation of modern care-seeking behavior remains a challenge [23]. A large number of children die without ever reaching a health facility. This is attributed to delays in seeking care by mothers. This delay affects children's health and leads to child health complications that make medical care ineffective [26, 27]. As a result, appropriate care-seeking behavior is an important parenting tool in preventing preventable morbidity and mortality in children [10].

To meet the Sustainable Development Goal (SDG) target one on under-five mortality by 2030, reducing the number of under-5 deaths by 10 million between 2017 and 2030, rapid improvement is needed to raise community awareness of modern health-seeking behavior [28]. Many programmes, such as Integrated Management of Childhood Illness (IMCI), have been established in order to ensure that children receive adequate care during their illness by providing direction to the world child health community on the best ways to assist countries in ensuring child survival [29].

The importance of caregivers' ability to seek appropriate care for their children is one of the recommended key activities in the WHO's and UNICEF's global action plans for the control of pneumonia and diarrhea. In addition, WHO, using the Child Health and Nutrition Research Initiative (CHNRI) methodology, identified the investigation of barriers to healthcare seeking and healthcare access as the highest primary research priority for reducing mortality from childhood pneumonia worldwide [29].

Although Ethiopia has implemented many strategies and has universal access to improve standard management of childhood diarrhea, fever, and respiratory tract infections, the modern care-seeking behavior of caregivers for childhood illnesses remains low [17]. However, despite these efforts to promote child health, many mothers or caregivers do not seek medical care for their children [11].

The focus of most studies was on the level or factors influencing health-seeking behavior for children in a specific district or region of the country. There is no review of the literature on cumulative care-seeking behavior for both the three main infectious causes of childhood mortality in Ethiopia: acute respiratory tract infection, diarrhea, and fever. There is a need for a study that would consider the pooled level of seeking behavior and associated factors at national level. In addition, knowledge of the local context is important to understand some of the factors that influence care-seeking behavior. This study is aimed at filling this gap by examining mothers' health seeking behavior for sick children and associated factors in Ethiopia as a whole.

The information generated from this study will allow managers to design appropriate strategies to address gaps related to mothers' or caregivers' health seeking behavior for their sick under-five children. Therefore, this review is aimed at determining the percentage of caregivers' health seeking behavior with a child of less than 5 years who were able to recognize the signs and symptoms of acute respiratory tract infection, diarrhea, and fever in their child and sought healthcare from different types of healthcare providers and its determinant in Ethiopia.

## 2. Materials and Methods

### 2.1. Searching Strategy

The systematic review and meta-analysis were carried out in accordance with the Preferred Reporting Items for Systematic Reviews and Meta Analyses (PRISMA) guideline [30] (Supplementary file 1). We have reviewed published and unpublished data related to the level of mothers' or caregivers' healthcare-seeking behavior for childhood diarrhea, fever, and respiratory tract infections and associated factors in Ethiopia until September 30, 2021. Relevant studies have been identified through databases (Google Scholar, PubMed, CINHAL, ScienceDirect, HINARI, and gray literatures).

The key terms to retrieve the studies were (level OR magnitude OR prevalence OR proportion OR epidemiology) AND (mother OR caregiver) AND (healthcare seeking OR health seeking OR care seeking OR help seeking OR health behavior) And (common childhood illness OR diarrhea OR malaria OR fever OR pneumonia OR cough OR respiratory tract infection) AND (under five years OR less than five years OR less than 59 months) AND (determinant OR factor OR cause or associated factors) AND Ethiopia.

### 2.2. Selection of Studies

The titles and abstracts of retrieved studies have been reviewed for relevance, and the full-text versions of potentially relevant articles were then analyzed according to the inclusion criteria detailed below. Reference lists of all included studies were checked for additional references. To avoid selection bias, the literature was searched by two authors independently. All citations were imported into an electronic database (endnote).

### 2.3. Eligibility Criteria

#### 2.3.1. Inclusion Criteria

This review includes observational studies (cross-sectional, case-control, and cohort studies) with original quantitative data, sample sizes of more than 50 participants, literature in English, published and unpublished articles, and articles on mothers/caregivers with under-five children who have had a sign or symptom of childhood illness.

#### 2.3.2. Exclusion Criteria

Qualitative and primary studies that were not fully accessible were excluded.

### 2.4. Operational Definitions

Acute respiratory infection (ARI): all cases with coughing and breathing difficulties reported by mothers or caregivers within two weeks of the survey [22]

Diarrhea: defined as three or more loose or watery stools per day, or blood in the stool, as perceived and reported by mothers or caregivers within two weeks of the survey [22]

Fever: an increase in body temperature or a feeling of being overheated in the selected child, as perceived and reported by mothers or caregivers within two weeks of the survey [22]

Healthcare-seeking behavior: mothers' or caregivers' responses to signs and symptoms of illnesses to reduce severity and complications after recognizing the child's illness [22]

### 2.5. Outcome Measurement

The study has two main objectives. The first is to determine the pooled level of mothers' or caregivers' healthcare-seeking behavior for childhood diarrhea, fever, and respiratory tract infections in Ethiopia. It was calculated by dividing the total number of mothers with under five ill children (children with diarrhea, fever, and respiratory tract infection) by the total number of mothers included in the study (sample size) and multiplying by one hundred (100). The second objective is to estimate the pooled effects of each factor on health seeking behavior, and the odds ratio was calculated from the primary studies using Excel and Stata software.

### 2.6. Data Extraction

Two independent authors extracted all the necessary data using a standardized data extraction format prepared in Microsoft Excel. For the level of health seeking behavior, the data extraction format included the author, publication year, study design, region of the country, number of samples, screening tool used, response rate, and level of health seeking behavior. For associated factors, the data extraction format has been prepared in the form of a two-by-two table for each significant variable. Any disagreements between the authors have been solved through discussion and double extraction of the inconsistent data.

### 2.7. Quality Assessment

The Newcastle-Ottawa Scale for observational study quality assessment tool was adapted to assess the quality of the studies included in the review and meta-analysis [31]. Two authors independently evaluate the quality of the original articles using this assessment tool as a guideline. The tool has indicators consisting of three main parts: the first part has five components and assesses the methodological quality of each study; the second section examines the comparability of the studies; the third part measures the quality of the original articles with respect to their statistical analysis. Finally, articles of medium and high quality have been included for analysis. Disagreements of assessors have been settled by taking the mean score of their assessment results. In general, there is no any article excluded from this study due to poor quality.

### 2.8. Method of Data Analysis

Important data was extracted using Microsoft Excel format, and then, it was imported to Stata version 14.0 software for analysis. The characteristics of original articles have been described using texts, tables, and forest plots. The standard error of prevalence for each original article was calculated using the binomial distribution formula. Heterogeneity among the reported prevalence of studies was checked by using a heterogeneity *χ*^2^ test and *I*^2^ test. Publication bias has been examined by performing Egger's correlation and Begg's regression intercept tests at a 5% significant level. In addition, subgroup analysis was conducted based on the illness type and region of studies conducted and publication year to minimize the random variations between the point estimates of the primary studies.

## 3. Results

### 3.1. Results of the Literature Search

**Figure 1:**
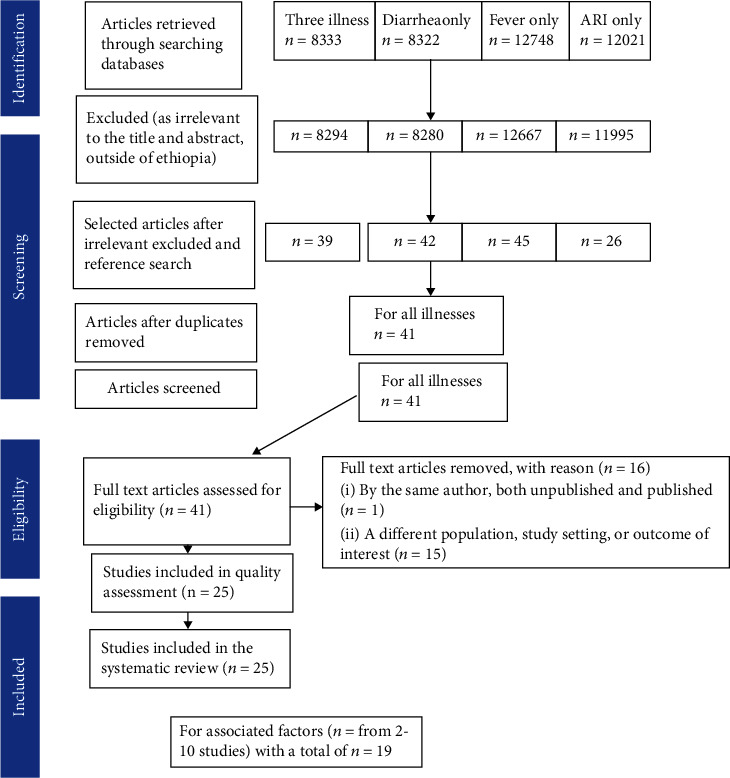
PRISMA statement presentation for systematic review and meta-analysis of mothers' or caregivers' healthcare-seeking behavior for childhood diarrhea, fever, and respiratory tract infections and associated factors in Ethiopia.

In the first step of our search, we retrieved 8322 studies for diarrhea, 12,748 for fever, 12021 for acute respiratory tract infection (ARI), and 8333 studies for the three childhood diarrhea, fever, and respiratory tract infections at the same time from different databases. Out of this scan, 8280, 12667, 11995, and 8294 retrieved studies were omitted for diarrhea, fever, ARI, and all three illnesses, respectively, via a step-by-step procedure as irrelevant to the title and abstract, outside of Ethiopia. Additionally, 152 articles were removed as duplicates for all types of illness. Hence, we read the full texts of 41 articles for all three illnesses and assessed their eligibility based on the preset criteria. About 15 studies were further excluded due to the differences in the study population and study settings, and one unpublished study was excluded because it was available online as both unpublished and published. Finally, 25 studies were found to be eligible and included in the systematic review and meta-analysis (Figure 1).

### 3.2. Study Characteristics

Of the 25 studies included in this review, all of them were observational and quantitative, two were facility-based cross-sectional [11, 32], one was longitudinal [33], and 22 were community-based cross-sectional. Out of 22 cross-sectional studies, nineteen were noncomparative [16–18, 20–24, 26, 27, 34–42], and three were comparative [15, 25, 43].

Based on the study setting, three of the studies were conducted in Addis Ababa [11, 17, 21], two in the Tigray region [18, 42], seven in the Amhara region [20, 22, 23, 25–27, 37], seven in the Oromia region [15, 32–35, 38, 43], one in the SNNP region [24], one in the Benishangul region [39], and four were conducted nationwide [16, 36, 40, 41]. Regarding the illness category, fifteen studies were conducted by including all the three childhood diarrhea, fever, and respiratory tract infections at the same time [15, 17, 20–25, 27, 32, 33, 36–38, 42], four were for diarrhea only [18, 21, 26, 43], three were for fever only [34, 35, 39], and three were for ARI only [16, 40, 41] (Table 1).

Of the 25 studies, 19 have factors associated with mothers' health-seeking behavior. Twelve studies identified factors for three illnesses [11, 15, 17, 19, 20, 23–25, 27, 36–38], four for diarrhea [18, 21, 26, 43], two for fever [35, 39], and one for ARI [16] (Table 2).

### 3.3. Meta-Analysis

**Figure 2:**
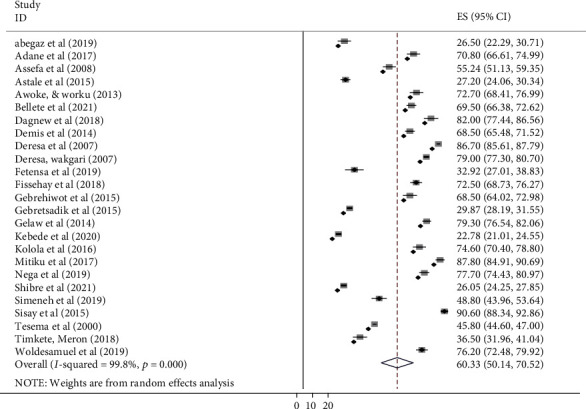
Forest plot for the pooled level of mothers' or caregivers' healthcare-seeking behavior for childhood diarrhea, fever, and respiratory tract infections in Ethiopia.

**Figure 3:**
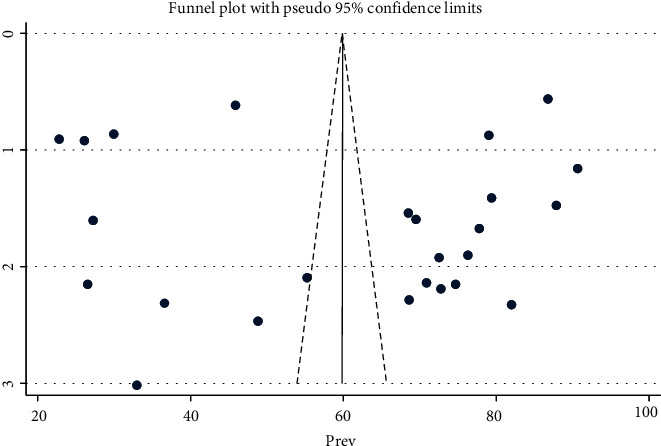
Funnel plot to show publication bias of studies for the pooled level of mothers' or caregivers' healthcare-seeking behavior for childhood diarrhea, fever, and respiratory tract infections in Ethiopia.

In this review, the overall pooled level of mothers' or care givers' health seeking behavior for childhood diarrhea, fever, and respiratory tract infections was 60.33% (95% CI: 50.14-70.52) with a range from 22.78% (Kebede et al., 2020) to 90.6% (Sisay et al., 2015) (Table 3). A random effect model was used, and the result of the heterogeneity test was (*I*‐squared = 99.8%, *P* < 0.01) (Figure 2). In addition, heterogeneity was checked for subgroups (each illness) and ranged from *I*‐squared = 76.8-99.7%, with *P* < 0.01. So, it shows that there is a heterogenicity between studies. With respect to publication bias, it was assessed by using a funnel plot, which was found to be a symmetrical distribution of included studies (Figure 3) and by applying Egger's test (*P* = 0.97). Both methods revealed the absence of publication bias among studies.

### 3.4. Subgroup Analysis

In our meta-analysis, we performed a subgroup analysis based on the regions where the studies were conducted, types of illness, publication years, and sample size. Accordingly, nationwide studies showed the lowest pooled level of health seeking behavior (HSB) (29.45% (95% CI: 26.11-32.79)), where as a study conducted in the Tigray region had the highest level of health seeking behavior (HSB) (74.36% (95% CI: 70.74-77.99)). Regarding types of illness, the lowest HSB was observed in acute respiratory infection (29.54% (95% CI: 24.36-34.71)) and the highest in fever (84.45% (78.9-89.99)). The level of HSB was almost similar in studies published before and after 2015. In addition, studies with a large sample size (>1000) have a lower pooled level of HSB at 44.25 percent (95% CI: 24.49–64.02). Furthermore, the result of sensitivity analysis revealed that there is no single study that affects the pooled level of health seeking behavior (Table 4).

### 3.5. Factors Associated with Mothers' or Caregivers' HSB

**Figure 4:**
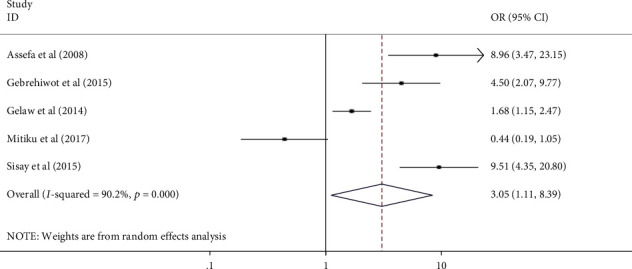
Forest plot showing the pooled odds ratio of residence in association with mothers' HSB for childhood diarrhea, fever, and respiratory tract infections in Ethiopia (2021).

**Figure 5:**
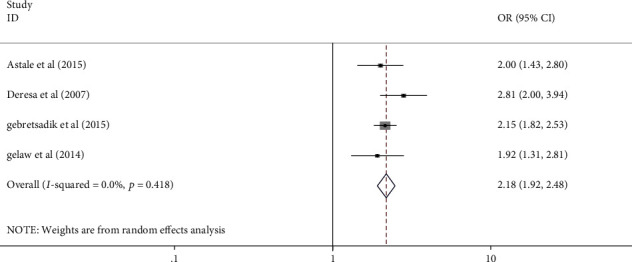
Forest plot showing the pooled odds ratio of wealth index in association with mothers' HSB for childhood diarrhea, fever, and respiratory tract infections in Ethiopia (2021).

**Figure 6:**
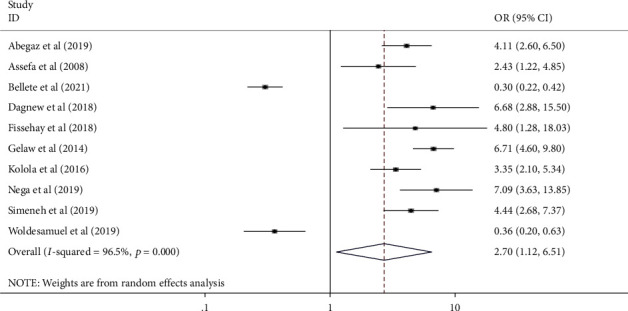
Forest plot showing pooled odds ratio of perceived severity of illness in association with mothers' HSB for common childhood illness in Ethiopia (2021).

**Figure 7:**
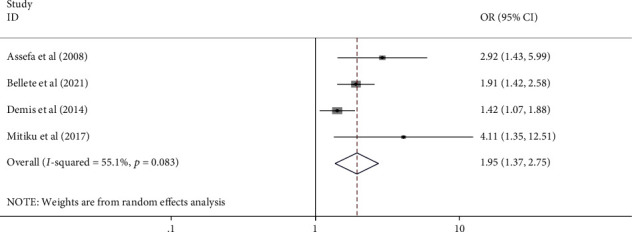
Forest plot showing pooled odds ratio of mothers' knowledge on illnesses in association with HSB for common childhood illness in Ethiopia (2021).

All authors have analyzed the potential factors of mothers' or caregivers' health-seeking behavior for childhood diarrhea, fever, and respiratory tract infections in Ethiopia using data from nineteen [18] studies. Variables that have been indicated as significant at least in two studies were included in the analysis. From those studies, child sex, mothers' educational status, residence, wealth index, perceived severity of illnesses, and knowledge of mothers for the illness were included in the study. Finally, residence, wealth index, perceived severity of illness, and knowledge of mothers or care givers for the illness were significantly associated with health seeking behavior for childhood diarrhea, fever, and respiratory tract infections (Table 5 and Figures 4–7).

Mothers/care givers in urban areas seek healthcare for childhood diarrhea, fever, and respiratory tract infections more than three times as frequently as their counterparts (AOR: 3.055, 95% CI: 1.11–8.39) (Figure 4). Mothers with a high wealth index seek care for their children's illnesses more than twice as often as mothers with a low wealth index (AOR: 2.18, 95% CI: 1.92-2.48) (Figure 5). Mothers who perceived the illness as severe were 2.7 times more likely to seek care for their children's illness than their counterpart (2.7 (95% CI: 1.12–6.51)) (Figure 6). Furthermore, mothers with good knowledge of childhood illnesses were nearly twice as likely as those with poor knowledge (1.95 (95% CI: 1.37–2.75)) (Figure 7).

## 4. Discussion

Treatments for childhood diarrhea, fever, and respiratory tract infections are usually very effective if healthcare is sought promptly. The challenge is to implement on-going programs such as IMNCI/ICCM that could educate caregivers and facilitate appropriate healthcare-seeking behavior.

This study showed that the overall prevalence of mothers'/care givers' health seeking behavior for childhood diarrhea, fever, and respiratory tract infections in Ethiopia was 60.33%, with a range from 22.78% to 90.6%. The lowest HSB was observed in acute respiratory infection (29.54%) and the highest in fever (84.45%). This study was lower than a study conducted in some developing countries in which a median of 73.0% of caregivers sought care from a healthcare provider when their child was suffering from those illnesses [10], and in Tanzania, 85% of children with suspected pneumonia were taken for care. In addition, 85.5% of women in SSA sought healthcare for childhood illnesses, with the highest and lowest prevalence in Gabon (75.0%) and Zambia (92.6%), respectively [13]. This difference in the prevalence of health-seeking for childhood diseases across countries could be a reflection of differences in socio-cultural and economic factors across the regions.

This review revealed that mothers/care givers residing in urban areas seek healthcare more than three times as often for childhood diarrhea, fever, and respiratory tract infections as their counterparts (AOR 3.055 (95% CI: 1.11–8.39)). This finding is supported by studies conducted in Kenya, Malawi, Nigeria, Honduras, and other developing countries, and they found that caregivers in urban areas were more likely to seek care than those in rural locations [10, 13, 44–48]. This higher healthcare-seeking behavior among urban mothers and care givers might be due to the availability and accessibility of health services in urban areas and the good knowledge of mothers and caregivers towards their child's illness.

According to our findings, mothers with a high wealth index seek medical attention for their child's illness more than twice as often as mothers with a low wealth index (2.18 (95% CI: 1.92-2.48)). The finding of this study is in line with studies in Kenya, Zambia, Burkina Faso, Mali, India, and other sub-Saharan African countries [10, 44, 49–52], and they cited cost as a reason for not seeking care for children with illness. Another study in Nigeria showed that income or finance is a strong determinant for mothers' decisions to seek care for their children [7]. In addition, children from the richest households were 9.5 times more likely to be brought for care than children from the poorest households in Tanzania [9]. Household income was significantly associated with healthcare seeking up to certain threshold levels [53]. That evidence indicates that income plays a significant role in mothers' healthcare-seeking behavior for their children. However, one study from Kenya [54] commented on the low rates of appropriate care seeking despite healthcare being free of charge at the point of care in the study locations.

This review result revealed that mothers who perceived severe illness were 2.7 times more likely to seek care for their children's illness than their counterpart (AOR: 2.7, 95% CI: 1.12–6.51). This result is supported by three studies [10, 55, 56] which found that the more severe caregivers perceived the child's illness to be, the more likely they were to seek care. This is also supported by a study conducted in Malawi in which when illness was perceived to be severe, health-seeking behavior also increased by 2.4 folds [57]. One study in Nairobi showed that perception of illness severity was strongly associated with healthcare seeking [53]. The above studies clearly indicate to us that mothers or caregivers sought healthcare for their ill children when the illness was worse.

In addition, mothers who had good knowledge of childhood illnesses were around 2 times more likely to seek healthcare than those with poor knowledge (1.95 (95% CI: 1.37–2.75)). This finding is supported by a study conducted in sub-Saharan African countries such as the Democratic Republic of the Congo and Nigeria, which said that there was a positive association between knowledge of mothers or caregivers and care seeking for suspected pneumonia children [58]. The above studies tell us how much the knowledge of mothers' or caregivers' matters in seeking healthcare for their ill children.

### 4.1. Limitations of the Study

It is unlikely that this review has identified all relevant studies because we did not search all the gray literature, the search strategy was carried out using English search terms only, and studies without an English abstract were not reviewed for inclusion in the review. The variations in study designs, illness definitions, and healthcare provider categories between studies may affect the comparability of the studies' results. A further weakness of the included studies arises from the fact that there is considerable overlap in the clinical presentation between the three illnesses included in this review as well as other childhood illnesses. It is, therefore, likely that a proportion of participants in the included studies were misdiagnosed.

## 5. Conclusion

This review suggested that the overall pooled prevalence of mothers' or care givers' health-seeking behavior for childhood diarrhea, fever, and respiratory tract infections was 60.33%. Residence, wealth index, perceived severity of illness, and knowledge of the illness by mothers were the significant factors. Therefore, providing interventions by considering the above factors will improve the overall seeking behavior. Health education and behavior change communication activities about early detection of childhood illnesses and the importance of seeking early treatment can be conducted with rural mothers/care givers.

## Figures and Tables

**Table 1:** Characteristics of studies on care seeking behavior based on some criteria.

Groups	Three illness	Diarrhea	Fever	ARI	Total
Location					
AA	2	1	0	0	3
Oromia	4	1	2	0	7
Amhara	6	1	0	0	7
South	1	0	0	0	1
Benishangul	0	0	1	0	1
Tigray	1	1	0	0	2
Nationwide	1	0	0	3	4
Date of publication					
≤2015	8	1	2	1	12
>2015	7	3	1	2	13
Sample size	6	2	0	0	8
≤500					
501-1000	6	2	1	0	9
>1000	3	0	2	3	8

**Table 2:** A summary of studies retrieved from literature search on the level of mothers' or caregivers' healthcare-seeking behavior for childhood diarrhea, fever, and respiratory tract infections and associated factors in Ethiopia (2021).

Illness category	Total number of articles retrieved	Total number of articles before duplicate removed	Selected for full text review	Included in the review	Factors with HSB
Three in one	8333	39		15	12
Diarrhea only	8322	42		4	4
Fever only	12748	45		3	2
ARI only	12021	26		3	1
All illnesses			41	25	19

**Table 3:** Summary table on the level of mothers' or caregivers' healthcare-seeking behavior for childhood diarrhea, fever, and respiratory tract infections in Ethiopia included in the systematic review and meta-analysis (2021).

Region/city	Location	Author	Publication year	Quality assessment	Total sample	Response rate	Prevalence (95% CI)
Addis Ababa	AA	Abegaz et al.	2019	8	422	100	26.5 (22.29-30.71)
	AA	Adane et al.	2017	7	472	95.8	70.8 (66.6-74.99)
	AA	Bellete et al.	2021	8	875	95.31	69.5 (66.38-72.63)
Oromia	Dera	Assefa et al.	2008	7	612	92.0	55.2 (51.1-59.35)
	Adami Tulu	Deresa et al.	2007	6	3873	95.7	86.7 (85.6-87.79)
	Wolega	Fetensa et al.	2019	8	243	100	32.9 (27.0-38.83)
	Arisi	Gebrehiwot et al.	2015	7	434	95.2	68.5 (64.0-72.98)
	Jeldu	Kolola et al.	2016	8	422	97.5	74.6 (70.39-78.8)
	Jimma	Tesema et al.	2000	8	8161	81.25	45.8 (44.6-46.99)
	Adami Tulu	Deresa, Wakgari	2007	7	2253	97.0	79.0 (77.3-80.7)
Amhara	Dangla	Dagnew et al.	2018	8	273	100	82.0 (77.4-86.56)
	Bahirdar	Awoke, Worku	2013	8	422	98.34	72.7 (68.4-76.99)
	Bure	Gelaw et al.	2014	8	886	93.3	79.3 (76.54-82.06)
	Gondar	Kebede et al.	2020	7	2226	96.95	22.78 (21.0-24.55)
	Dangla	Nega et al.	2019	7	624	100	77.7 (74.4-80.97)
	Aneded	Simeneh et al.	2019	8	410	100	48.8 (43.96-53.64)
	Ensaro	Sisay et al.	2015	7	641	99.8	90.6 (88.34-92.86)
South region	Shashogo	Demis et al.	2014	8	908	99.9	68.5 (65.478-71.5)
Benishangul-Gumuz	Mandura	Mitiku et al.	2017	7	503	97.6	87.8 (84.9-90.69)
Tigray	Mekelle	Fissehay et al.	2018	8	540	100	72.5 (68.7-76.27)
	Shire	Woldesamuel et al.	2019	7	504	100	76.2 (72.48-79.92)
Nationwide	Nationwide	Astale et al.	2015	8	11030	100	27.2 (24.06-30.34)
	Nationwide	Gebretsadik et al.	2015	8	11645	100	29.87 (28.19-31.5)
	Nationwide	Timkete, Meron	2018	7	10641	100	36.5 (31.96-41.04)
	Nationwide	Shibre et al.	2021	8	2284	100	26.05 (24.25-27.8)
*D* + *L* pooled ES							60.33 (50.14-70.5)

**Table 4:** Subgroup analysis for the level of mothers' or caregivers' healthcare-seeking behavior for childhood diarrhea, fever, and respiratory tract infections in Ethiopia.

Criteria	Number of studies	Prevalence rate (95% CI)	*I* ^2^ (*P* value)
Location			
AA	3	55.62 (28.52-82.72)	99.3% (*P* < 0.01)
Oromia	7	63.31 (46.68-79.95)	99.8% (*P* < 0.01)
Amhara	7	67.69 (43.39-91.99)	99.8% (*P* < 0.01)
South region	1	68.5 (65.48-71.52)	—
Benishangul	1	87.8 (84.9-90.69)	—
Tigray	2	74.36 (70.74-77.99)	46.7% (*P* = 0.17)
Nationwide	4	29.45 (26.11-32.79)	86.5% (*P* < 0.01)
Types of illness			
Three illness	15	58.34 (46.37-70.34)	99.7% (*P* < 0.01)
Diarrhea	4	72.56 (68.52-76.59)	76.8% (*P* < 0.01)
Fever	3	84.45 (78.9-89.99)	96.7% (*P* < 0.01)
ARI	3	29.54 (24.36-34.71)	88.6% (*P* < 0.01)
Date of publication			
≤2015	12	60.84 (46.69-74.99)	99.8% (*P* < 0.01)
>2015	13	59.86 (44.79-74.92)	99.7% (*P* < 0.01)
Sample size			
≤500	8	59.64 (45.46-73.82)	98.7% (*P* < 0.01)
501-1000	9	75.32 (68.41-82.23)	97.8% (*P* < 0.01)
>1000	8	44.25 (24.49-64.02)	99.9% (*P* < 0.01)

**Table 5:** A summary of factors associated with mothers' or caregivers' healthcare-seeking behavior for childhood diarrhea, fever, and respiratory tract infections in Ethiopia.

Variables	Responses	Pooled OR (95% CI)	*P* value
Child sex	Male Female	1.26 (0.95-1.66)	*P* = 0.11
Mothers' education	No formal education Formal education	0.59 (0.28-1.25)	*P* = 0.17
Residence	Urban Rural	3.06 (1.11-8.39)	*P* = 0.03
Wealth index	High Low	2.18 (1.92-2.48)	*P* < 0.01
Perceived severity of illness	Severe not Severe	2.7 (1.12-6.51)	*P* = 0.03
Knowledge of mothers for the illness	Good Poor	1.95 (1.37-2.75)	*P* < 0.01

## Data Availability

The data used to support the findings of this study are available from the corresponding author upon request.

## Abbreviations

HSB: Health seeking behavior
IMNCI: Integrated Management of Newborn and Childhood Illness
ICCM: Integrated Community Case Management
NOS: Newcastle-Ottawa Scale
PRISMA: Preferred Reporting Items for Systematic Reviews and Meta-Analyses
SPSS: Statistical Package for Social Science
WHO: World Health Organization.

## Supplementary Materials

Supplementary Materials The systematic review and meta-analysis were carried out in accordance with the Preferred Reporting Items for Systematic Reviews and Meta Analyses (PRISMA) guideline [31] (Supplementary file 1).
